# Leptotrichia trevisanii bacteremia in a woman with systemic lupus erythematosus receiving high-dose chemotherapy

**DOI:** 10.1186/s12879-018-3495-9

**Published:** 2018-12-14

**Authors:** Hongyan Hou, Zhongju Chen, Lei Tian, Ziyong Sun

**Affiliations:** Department of Laboratory Medicine, Tongji Hospital, Tongji Medical College, Huazhong University of Science and Technology, Jiefang Road 1095#, Wuhan, 430030 China

**Keywords:** *Leptotrichia trevisanii*, Bacteremia, SLE

## Abstract

**Background:**

*Leptotrichia* species are aerotolerant, Gram-negative fusiform bacteria. Cases of bacteremia caused by *Leptotrichia trevisanii* in immunocompromised patients have been rarely reported.

**Case presentation:**

A 33-year-old female with systemic lupus erythematosus (SLE) was admitted to the department of rheumatology with bleeding from a mucosal ulcer. One month previously, she had visited our hospital and begun to receive methotrexate therapy, but mis-dosed for nearly 1 month at home. Methotrexate toxicity resulted in a severe oral ulcer and bone marrow suppression. On day-7 of hospital admission, she developed a fever, and Gram-negative rods (*Leptotrichia trevisanii*) were detected in blood cultures. She was diagnosed with methotrexate poisoning followed by *L. trevisanii* bacteremia. After antibiotic and detoxification therapy, she recovered from bacteremia, and the oral ulcer and bone-marrow suppression improved obviously.

**Conclusions:**

This is the first reported case of *Leptotrichia trevisanii* bacteremia in a SLE patient who took mis-dosed an immunosuppressant and had an oral mucosal lesion.

## Background

*Leptotrichia* species are aerotolerant, Gram-negative fusiform bacteria that can inhabit the oral cavity, intestinal tract and genitalia [1]. *Leptotrichia* species have been implicated in periodontal disease, endocarditis, chorioamnionitis and arthritis, especially in immunocompromised patients [2–4]. *Leptotrichia trevisanii* bacteremia has been reported in patients with acute myeloid leukemia [5, 6], myelodysplastic syndrome [7] and febrile neutropenia [8]. Here, we describe a case of bacteremia due to *L. trevisanii* in a female with systemic lupus erythematosus (SLE) and methotrexate poisoning.

## Case presentation

A 33-year-old female was diagnosed with SLE 3 years previously in another hospital. She visited our hospital in May 2018 due to pain in lower back and began to take methotrexate. Advice from her physician was methotrexate (10 mg) once a week, but she made the mistake of taking this dose every day. She developed severe bleeding from an ulcer in the oral mucosa, and was admitted to our hospital 1 month later. She was diagnosed with SLE and methotrexate poisoning.

**Fig. 1:**
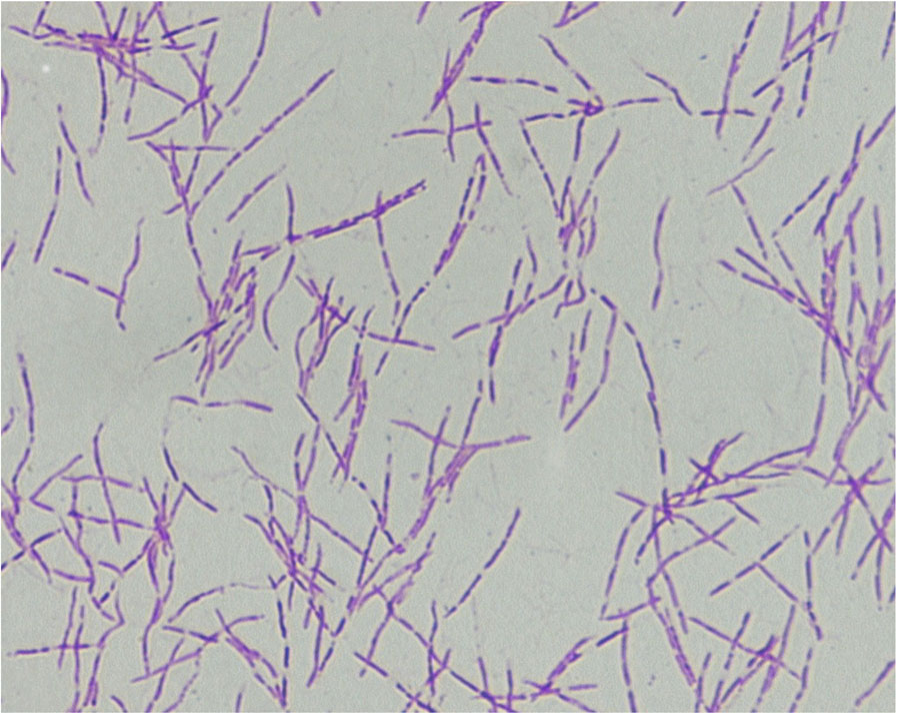
Gram stain preparation of the isolate colony in anaerobic condition (grown on a blood agar plate). Gram stain × 1,000

**Fig. 2:**
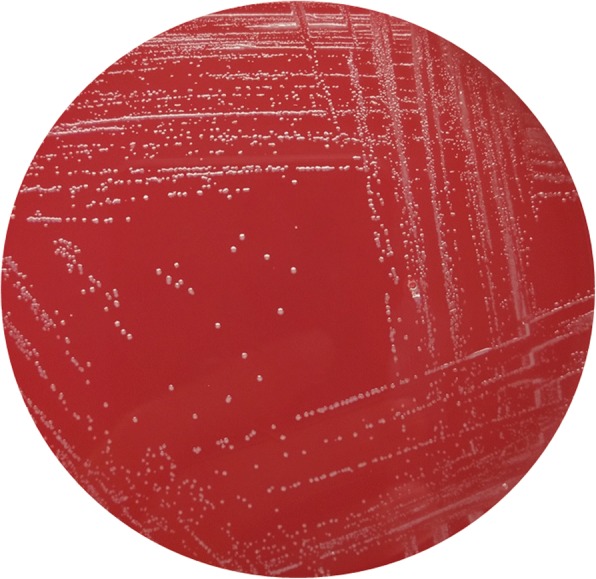
The form of colonies on a blood agar plate

**Fig. 3:**
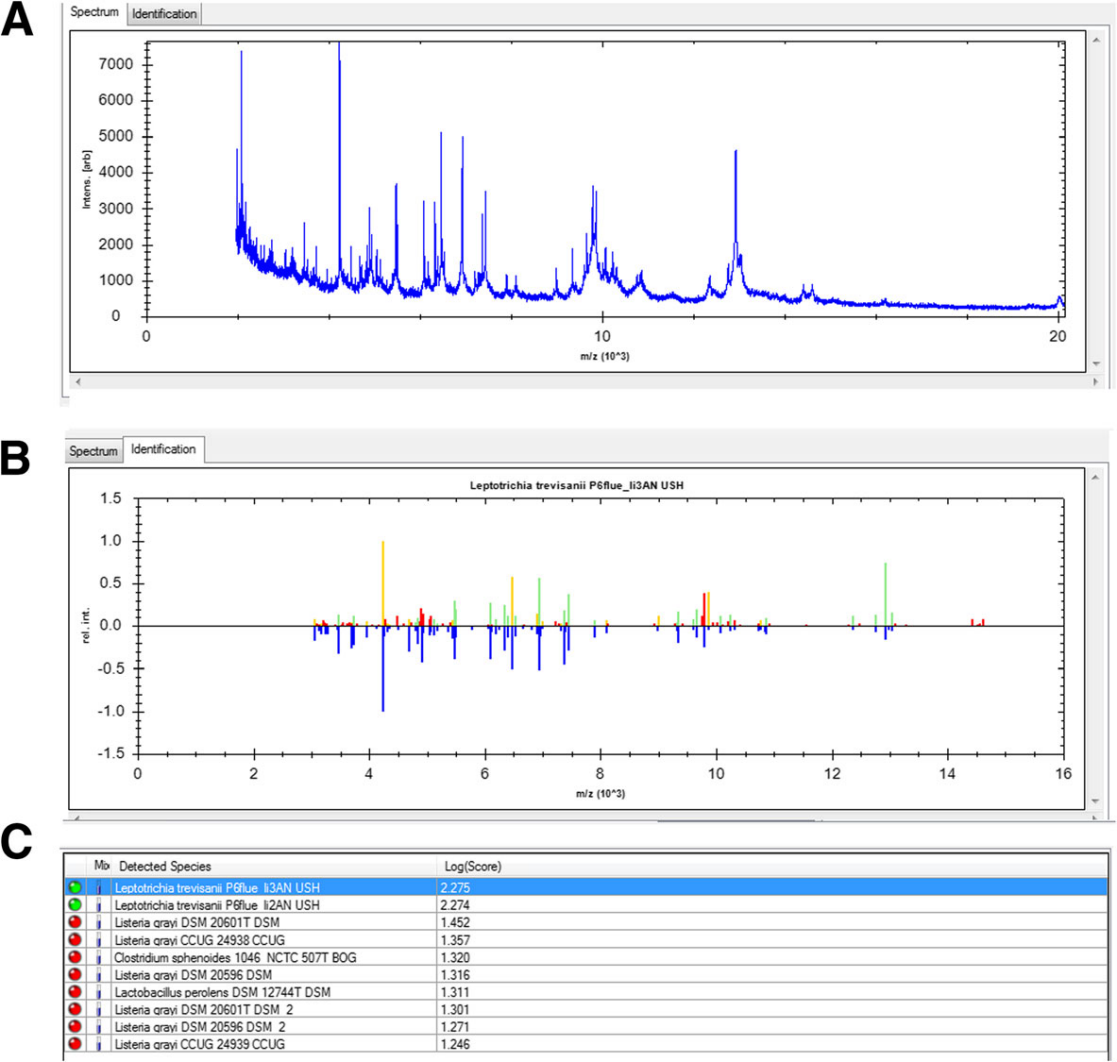
Identification of *L. trevisanii* by MALDI-TOF MS **a** The spectrum of L. *trevisanii*. **b**, **c** Identification result of L. *trevisanii*

Even though she had stopped using methotrexate, her white blood cell (WBC) count was 1.67 × 10^9^/L, red blood cell (RBC) count was 3.02 × 10^9^/L, hemoglobin level was 119 g/L, and platelet count was 66 × 10^9^/L, which suggested bone suppression. On day-7 of hospital administration, the patient developed fever (38.3 °C) and infection was suspected. Laboratory analyses revealed a level of C-reactive protein (CRP) of 19.19 mg/L, lactate dehydrogenase (LDH) of 5 U/L, aspartate transaminase (AST) of 8 U/L, alanine transaminase (ALT) of 91 U/L, and erythrocyte sedimentation rate (ESR) of 23 mm/h. Blood samples were taken and empirical therapy with metronidazole and cefoperazone/tazobactam was started because of fever with oral mucositis. At day- 11 of hospital admission, spindle-shaped Gram-negative rods were isolated from one of four blood samples (Fig. 1). After 24 h of incubation in an anaerobic environment with 5% CO_2_, growth on blood agar showed light yellow-pigmented colonies that were smooth, gray, catalase-positive and oxidase-negative (Fig. 2). The organism was identified as *Leptotrichia trevisanii* by matrix-assisted laser desorption ionization- time of flight-mass spectrometry (MALDI-TOF-MS) undertaken on a Biotyper system (Bruker, Bremen, Germany) (Fig. 3). In addition, the organism was confirmed as *L. trevisanii* using 16S ribosomal RNA (rRNA) gene sequencing as described previously [9], which matched 98% with the *L. trevisanii* strain (NR 029805.1). Therefore, the patient was diagnosed with *L. trevisanii* bacteremia and received the therapy mentioned above continuously.

Next, we collected samples of the ulcer in the oral mucosa using a swab for anaerobic culture but *L. trevisanii* was not isolated, which might have been caused by exposure to an aerobic environment. After 10 days of treatment, the fever disappeared and the mucositis improved obviously. Routine blood analyses revealed the WBC count to be 11.19 × 10^9^/L, RBC count to be 3.7 × 10^12^/L, hemoglobin level to be 110 g/L and platelet count to be 256 × 10^9^/L, suggesting that bone-marrow suppression was also restored. Therefore, sepsis had resolved and there was no recurrence of bacteremia.

## Discussion

*Leptotrichia* is one of six genera in the family *Fusobacteriaceae* in the phylum *Fusobacteria* [1]. The genus *Leptotrichia* contains a wide variety of species,such as *L. buccalis, L. trevisanii, L. amnionii, L. goodfellowi, L. hofstadii, L. shahii, L. wadei, and L. hongkongensis* [1, 10]. *Leptotrichia* species are anaerobic Gram-negative rods that inhabit the oral cavity, gastrointestinal tract, urogenital system and female genital tract of humans [11, 12].

Previous studies have shown that *Leptotrichia* species can be isolated as pathogens in endocarditis, periodontitis, chorioamnionitis, abscesses as well as wound infections after dog bites [4, 11]. Bacteremia caused by *Leptotrichia* species are rarely isolated from immunocompromised patients (e.g, those suffering from hematologic malignancies, cancer or acquired immune deficiency syndrome) [5, 11, 13, 14]. *Leptotrichia* species are not prevelant, but infection can be severe in immunosuppressed patients. Identification of *Leptotrichia* species can be difficult, and is likely to remain an underappreciated cause of anaerobic bloodstream infection due to the inherent difficulties associated with conventional laboratory assays [15]. Moreover, the clinical signs of infection with *Leptotricha* species are not specific because fever can occur in other Gram-negative bacterial infections. Therefore, misdiagnoses of infections due to *Leptotrichia* species with infections by *Fusobacterium* species*, Capnocytophaga* species *or Lactobacillus* species or the problems due to culture can obscure the clinical importance of *Leptotrichia* species. However, MALDI-TOF-MS and 16S rRNA gene sequencing can be employed to identify *Leptotrichia* species. Both of these methods are reliable and feasible for identification of some anaerobic species in microbiology laboratories that are difficult to identify using traditional phenotypic systems [6, 16]. Therefore, routine use of these new methods allows recongnition of more *Leptotrichia* isolates, which could also provide evidence for effective and timely therapy for patients.

*L. trevisanii* was described first by Tee et al. in 2001 [6]. *L. trevisanii* grow best in anaerobic environments, but they can grow readily aerobically with 5% CO_2_ on blood agar, and are recongized as the most aerotolerant species of this genus [1, 11]. On blood agar, colonies appear smooth, gray, with dark central spots, measuring ~ 2 mm. Gram stain reveals unsporulated, elongated, fusiform Gram-negative bacilli. *L. trevisanii* has been reported in bacteremia secondary to mucositis, periodontitis, abscesses, and catheter-related bloodstream infections as well as in patients: with hematologic malignancies; who have undergone bone transplantation; with solid tumors undergoing chemotherapy; with febrile neutropenia [5–8, 16–18]. Particullarly, patients who have undergone and hematopoietic stem-cell transplantation and who received high-dose chemotherapy, *Leptotrichia* species have been identified as major pathogens of bacteremia [12]. Chorioamnionitis and pelvic inflammatory diseases have also been reported to be caused by *L. trevisanii* [19, 20]. Therefore, *Leptotrichia* species as emerging pathogens can cause *Leptotrichia* anaerobic bloodstream infection in immunocompromised patients.

This is the first case of *L. trevisanii* bacteremia in a SLE patient diagnosed with methotrexate poisoning because of mis-dosing. Subsequently, she developed severe oral mucositis accompanied by neutropenia. Methotrexate poisoning is very rare, so we recommended that she take immunosuppressants according to the advice of her physician. Furthermore, not only patients with hematologic malignancies but also people with autoimmune diseases who take immunosuppressants (with the attendant loss of a normal mucosal barrier in oral or lower gastrointestinal tracts) should be considered as high-risk populations for *Leptotrichia* infection. Therefore, immunosuppression with severe mucositis and neutropenia are the primary risk factors for *L. trevisanii* bacteremia.

## Conclusion

In conclusion, this is the first case of *L. trevisanii* bacteremia in a SLE patient with methotrexate poisoning. This species can be identified by MALDI-TOF- MS rapidly, reliably, and cost-effectively, which enables early appropriate antibiotic treatment and prevention of complications. Opportunistic pathogens and oral anaerobes, including *L. trevisanii,* should be screened completely, not limited to conventional agents, in immunocompromised patients who have oral mucositis.

## Abbreviations

ALT: Alanine aminotransferase
AST: Aspartate transaminase
CRP: C reaction protein
ESR: Erythrocyte sedimentation rate
LDH: Lactate dehydrogenase
MALDI-TOF MS: Matrix-assisted laser desorption ionization time of flight spectrometry
RBC: Red blood cell
rRNA: Ribosomal RNA
SLE: Systemic lupus erythematosus
WBC: White blood cell
